# Comparative efficacy on dogs of a single topical treatment with fipronil/(S)-methoprene or weekly physiological hygiene shampoos against *Ctenocephalides felis* in a simulated flea-infested environment

**DOI:** 10.1051/parasite/2012192153

**Published:** 2012-05-15

**Authors:** F. Beugnet, J. Fourie, K. Chalvet-Monfray

**Affiliations:** 1 Merial 29, avenue Tony Garnier 69007 Lyon France; 2 ClinVet International (Pty) Ltd., Uitsig Road, Bainsvlei Bloemfontein 9338 Republic of South Africa; 3 Biomathematics, VetAgroSup, Veterinary Faculty of Lyon 1, avenue Bourgelat 69280 Marcy L’Étoile France

**Keywords:** dog, flea, simulated infestation, fipronil/(S)-methoprene, shampoo, chien, puce, système d’infestation continue, fipronil/(S)- méthoprène, shampooing

## Abstract

Flea infestations of pets continue to persist due to the lack of knowledge of flea biology and ecology. It is not unusual that pet owners believe regular hygiene, such as shampooing their dogs can replace regular insecticidal treatment. The objective of this study was to compare in a flea simulated environment, modelling exposure similar to that found in a home, that the use of regular physiological shampoo does not control fleas adequately when compared to a long acting topical formulation. Three groups of six dogs were formed: one untreated control group, one group treated monthly with the topical formulation of fipronil/(S)-methoprene, and a third group treated weekly with a hygiene shampoo. All dogs were infested with adult unfed *Ctenocephalides felis* fleas (200 ± 5) on Days -28 and -21. Each animal’s sleeping box was fitted with a plastic cup mounted to the inside roof of the box. The sleeping bench of each animal was covered with a carpet to accommodate flea development. The dogs were maintained in their kennels throughout the study. In order to maintain the environmental flea challenge, *C. felis* pupae (100 ± 5) were placed in the plastic cup in each animal’s sleeping box on Days -14, -7, 0, 7, 14, 21, 28, 35 and 42. The dogs were combed and fleas counted weekly on Days -1, 3, 10, 17, 24, 31, 38, 45, and 51. The fleas were placed immediately back on the dogs. On Day 60, fleas were counted and removed. Flea infestations in the untreated control group at each count averaged between 46.2 and 74.2 fleas throughout the study. The average number of fleas infesting dogs was significantly different (p < 0.05) between the untreated and the two treatment groups and between the two treatment groups at all counts throughout the two months study (Days 3, 10, 17, 24, 31, 38, 45, 51 and 60). The efficacy was never below 99.1% in the fipronil/(S)-methoprene group, and efficacy in the shampoo group was never above 79.2%. Weekly shampooing in treatment group 3 was intentionally delayed after Day 42, to evaluate wether missing a weekly bath would affect the flea population. The Day 48 data indicate that forgetting or delaying a single weekly shampooing resulted in a clear increase in flea numbers and a significant decrease in efficacy from 68.2% to 34.8%. The fipronil(S)/methoprene treatment allowed a continuous control as demonstrated by the high efficacy against fleas, and also the number of flea-free dogs on seven of the nine weekly assessments, in spite of what was essentially a continuous flea challenge model.

## Abstract (fr)

Les infestations des carnivores domestiques par les puces restent fréquentes du fait d’une méconnaissance de la biologie et de l’écologie des puces. Il n’est pas rare que les propriétaires de chiens ou de chats soient persuadés qu’une hygiène régulière, en particulier des shampooings, remplace les traitements insecticides. L’objectif de cette étude était de comparer, dans un système de simulation d’infestation par les puces via l’environnement, que l’application hebdomadaire de shampooing physiologique ne contrôle pas les puces de façon adéquate comparée à l’application d’un topique insecticide mensuel. Trois groupes de chiens ont été formés, un non traité, un recevant une dose de la formulation topique de fipronil/(S)-méthoprène, et un troisième recevant un shampooing physiologique toutes les semaines. Tous les chiens ont été infestés par des puces *Ctenocephalides felis* (200 ± 5) aux jours - 28 et - 21. Chaque niche individuelle de repos était équipée à son plafond d’une boîte permettant d’y placer des cocons de puces. Le sol était couvert d’un tapis permettant aux oeufs et larves de puces d’évoluer. Les chiens ont été maintenus individuellement dans un box avec niche de repos pendant toute l’étude. Pour maintenir le niveau d’infestation, des pupes de *C*. *felis* (100 ± 5) ont été placées dans les boîtes au plafond des niches aux jours - 14, - 7, 0, 7, 14, 21, 28, 35 et 42. Tous les chiens étaient peignés et les puces comptées aux jours - 1, 3, 10, 17, 24, 31, 38, 45 et 51. Les puces étaient replacées sur les chiens après comptage sauf au jour 60, fin de l’étude. Les chiens du groupe contrôle non traité ont été infestés en moyenne par 46,2 à 74,2 puces tout au long de l’étude. Cette quantité était significativement différente avec les deux groupes traités (p < 0,05), mais aussi entre les deux groupes traités durant tous les comptages effectués au cours des deux mois (jours 3, 10, 17, 24, 31, 38, 45, 51 et 60). L’efficacité insecticide n’est jamais descendue en dessous de 99,1 % dans le groupe fipronil/(S)-méthoprène, et l’efficacité n’a jamais été supérieure à 79,2 % dans le groupe “shampooings”. Le shampooing du jour 42 a volontairement été omis pour évaluer l’impact d’un oubli dans le schéma hebdomadaire. Les comptages du jour 48 ont indiqué clairement qu’un shampooing oublié faisait chuter l’efficacité de 68,2 % à 34,8 %. Le traitement fipronil/(S)-méthoprène a permis un contrôle continu démontré par l’efficacité anti-puces de plus de 99 % durant toute l’étude, mais aussi par le nombre significativement différent de chiens sans puce observé au cours des comptages hebdomadaires, dans un système d’infestation continue.

## Introduction

Throughout the world, fleas, particularly the species *Ctenocephalides*, are the main ectoparasites of domesticated dogs and cats (Rust & Dryden, 1997). Flea infestations cause irritation to animals and humans and can lead to disorders, such as anaemia and dermatological problems. Repeated infestations of dogs and cats can contribute to the development of flea allergic dermatitis (FAD) as a result of hypersensitivity to components of flea saliva (Dryden & Rust, 1994). Rust & Dryden (1997) estimated that flea-related diseases are responsible for 50% of dermatological cases presented to veterinarians. Worldwide, *Ctenocephalides felis felis* (the cat flea) is the most common flea species affecting dogs and cats. It is also important to veterinary and public health as *C. felis* can be reservoirs and potential vectors for a variety of pathogens, including zoonotic agents. The cat flea is a known vector of *Bartonella henselae*, *Bartonella clarridgeiae*, and *Rickettsia felis*. In humans, these organisms can cause cat scratch disease, endocarditis, and cat flea typhus, respectively (Bradbury & Lappin, 2010; Dryden & Rust, 1994; Durden & Traub, 2002). Cat fleas also act as an intermediate host for *Dipylidium caninum*, the common tapeworm of dogs and cats, which also can be transmitted to humans (Guzman, 1984).

*Ctenocephalides felis felis* are capable of establishing and maintaining infestations inside homes, often to the point where they are classified as a household pest. Despite the increasing availability and use of a number of effective products, flea infestations are still wide-spread in essentially every country around the world. Epidemiological surveys of flea infestation of dogs and cats have been carried out in various countries (Alcaino *et al.*, 2002; Beck *et al.*, 2006; Chee *et al.*, 2008; Dryden et al., 2011; Durden *et al.*, 2005; Farkas *et al.*, 2009; Franc *et al.*, 1998; Gonzales *et al.*, 2004; Gracia *et al.*, 2007; Koutinas *et al.*, 1995; Xhaxhiu *et al.*, 2009).

Flea infestations of cats and dogs remain widespread and continue to persist due to the lack of knowledge of flea biology and ecology by some veterinarians, the lack of education provided to pet owners, and the ensuing lack of compliance with control measures (Dryden, 2009; Farkas *et al.*, 2009). The lack of understanding is reflected in the fact that it is not unusual for pet owners to believe regular hygiene, such as shampooing their dog, can replace regular insecticidal treatment. The objective of this study was to compare efficacy of the use of regular physiological shampoo with the efficacy of a long-acting topical formulation containing both fipronil and (S)-methoprene in a flea simulated environment, modelling exposure similar to that found in a home.

## Materials and methods

This study was a negative controlled efficacy study using a randomized block design where blocks were based on pre-treatment flea counts within sex.

### Animals and parasites

Three groups of six dogs were formed (Table 1: one untreated control group, one group treated monthly with the topical formulation of fipronil/(S)-methoprene (FRONTLINE^®^ Plus, Merial) and a third group treated weekly with a hygiene shampoo (oat meal extract 2%, glycerin 5%, chitosanide, foam base qsp 100%).

**Table 1.** Constitution of the three groups.

Group	Investigational material	Total dose volume	Route	Treatment Day	Total No. animals
1	Untreated	NA	NA	6
2	FRONTLINE^®^ Plus	Based on dog’s weight, pipettes S or M (respectively 0.67 or 1.34 mL), Based on label recommendation	Topical	Day 0 and Day 28	6
3	Physiological Oat meal extract shampoo	Shampoo based on manufacturer recommendation	Topical	Days 0, 7, 14, 21, 28, 35, 42 and 53	6

The fleas used in this study were from a colony known to be susceptible to all ectoparasiticides and were sourced from ClinVet, South Africa, where they are maintained in the laboratory by continuous passage on dogs and cats.

Eighteen healthy dogs, nine males and nine females, Beagles and mixed breeds, weighing less than 20 kg, were selected based on pre-infestation flea counts.

The dogs had not been treated with ectoparasiticides or insect growth regulators (either topical or systemic) for at least three months prior to the start of the study. All dogs had a physical examination on Day -32 to ensure a healthy status. Dogs that were debilitated or suffering from disease were unsuitable for inclusion in this study. Dogs presenting skin abnormalities were also excluded. All dogs were shampooed with a noninsecticidal shampoo on Day -32 before the start of the study, and subsequently infested one time with 100 (± 5) adult unfed *C. felis*. Fleas were counted 24 hours later on Day -31, pre-treatment, for selection purposes.

**Fig. 1.**
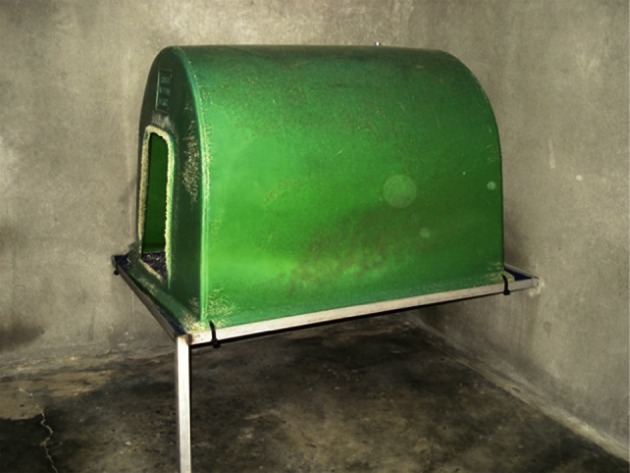
Sleeping box of each dog in its pen.

The dogs were held in individual pens that were part of an indoor animal unit, environmentally controlled for temperature (approximately 20 °C). A photoperiod of 12 hours light: 12 hours darkness was maintained throughout the study. Each dog pen was 2.05 m × 3.00 m. The pens were with solid brick walls, and no contact between dogs was possible. The study number, identification number, gender, group code and feed ration of the dog housed inside each pen was indicated on the outside of the pen. The pens had concrete floors to facilitate cleaning, and each was fitted with a sleep bench. A standard plastic kennel was fitted over the sleeping bench of each animal (Fig. 1).

Personnel involved in the flea counts were blinded as to treatment groups. All personnel with access to the treatment assignments were identified prior to initial treatment being administered and maintained blinding throughout the study.

### Treatment

The dogs from group 1 remained untreated. Dogs in treatment group 2 were treated with the appropriate pipette size of FRONTLINE^®^ Plus spot on Days 0 and 28. For treatment administration, the total volume was applied on one spot placed on the midline at the base of the neck. The hair was parted, until the skin is visible, then the tip of the pipette was placed just above the skin and squeezed to empty the contents directly onto the skin.

Dogs in treatment group 3 were shampooed per the study schedule (Table 1) with the appropriate volume of the physiological shampoo based on label recommendations of the manufacturer and then rinsed thoroughly with clear water.

### Study Design

**Fig. 2.**
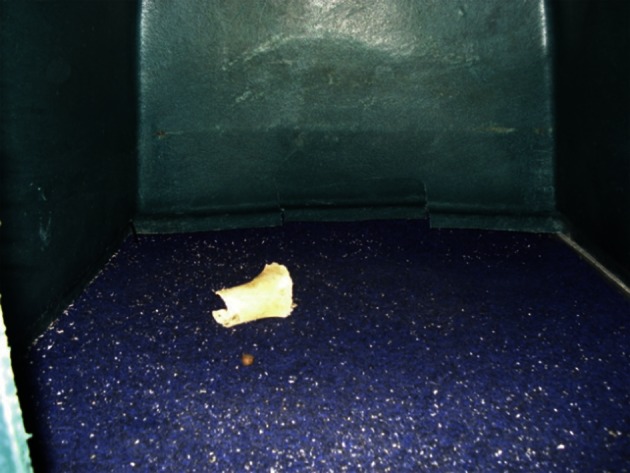
Carpet fitted inside each sleeping area.

**Fig. 3.**
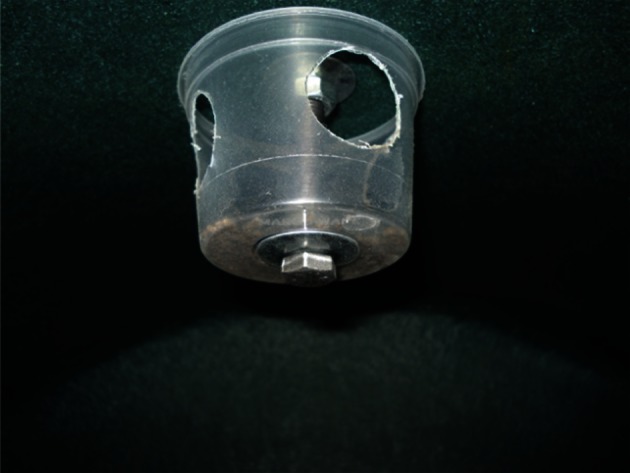
Plastic cup mounted to the inside roof of each sleeping box (where the flea pupae were deposited).

All dogs were placed in their pens on Day -32 for acclimatizing. They were infested with adult unfed *C. felis* fleas (200 ± 5) on both Days -28 and -21. Each animal’s sleeping box was fitted with a plastic cup, mounted to the inside roof of the box. The sleeping bench of each animal was covered with a carpet to accommodate flea development. The dogs were maintained in their kennels throughout the study, and each kennel was cleaned on a twice-daily basis, which reduced environmental flea contamination in the kennel. In order to maintain the environmental flea challenge levels more consistent with those seen in household infestations, *C. felis* pupae (100 ± 5) were placed in the plastic cup of each animal’s sleeping box on Days -14, -7, 0, 7, 14, 21, 28, 35 and 42, so newly emerged fleas would maintain a consistent environmental challenge during the length of the study (Figs 2, 3).

The dogs were combed and fleas were counted weekly on Days -1, 3, 10, 17, 24, 31, 38, 45, and 51. Then the fleas were immediately placed back on the dogs. On Day 60, fleas were counted and removed from all dogs.

### Variables and data analysis

The counts of live adult fleas were transformed to the natural logarithm of (count + 1) for calculation of geometric means by treatment group at each time point. Percentage reductions from the negative control mean (*i.e*., efficacy) were calculated using the formula [(C - T) / C] × 100, where C = geometric mean for the control group and T = geometric mean for the treated group. Arithmetic means were calculated.

The treatment groups were compared using the Kruskal-Wallis rank sum test for each study count day. Because all Kruskal-Wallis rank tests were significant, in a second step, a non-parametric multiple comparison test (Pairwise Wilcoxon Rank Sum Test) was performed for each date. A Fisher test was used to compare the numbers of flea-free dogs between the groups.

## Results

Flea infestations in the control group averaged between 46.2 and 74.2 fleas throughout the study (Table 2). Following the initial adult flea infestations of the dogs on Day -28 and Day -21, the only additional infestations of fleas resulted from the flea pupae placed in the kennel to simulate the level of flea exposure seen in home environments, especially in carpets.

**Table 2.** Statistical analysis of the flea counts.

Groups		Day -1	Day 3	Day 10	Day 17	Day 24	Day 31	Day 38	Day 45	Day 51	Day 60
Untreated control	Arithmetic mean	51	56	61.67	63.33	60	65.33	69.5	74.17	54	46.17
	Geometric mean^1^	38.73	45.34	55.27	56.14	53.35	59.88	63.2	66.63	40.68	31.16

Hygiene shampoo	Arithmetic mean	43.33	13.17	22.33	17.17	23.67	27.67	22.17	30.33	45.83	32.17
	Geometric mean^1^	39.98	9.43	14.72	12.88	17.13	19.51	14.32	20.91	26.54	19.8

FRONTLINE^®^ Plus	Arithmetic mean	57.17	0	0.33	0.67	0.17	0.33	0	0.5	0	0
	Geometric mean^1^	38.44	0	0.26	0.51	0.12	0.2	0	0.26	0	0

P (statistical difference at p < 0.05 between the 2 treatment groups)		NS	0.011	0.0035	0.0062	0.0044	0.004	0.0048	0.0051	0.0048	0.0047

1 Geometric mean

**Fig. 4.**
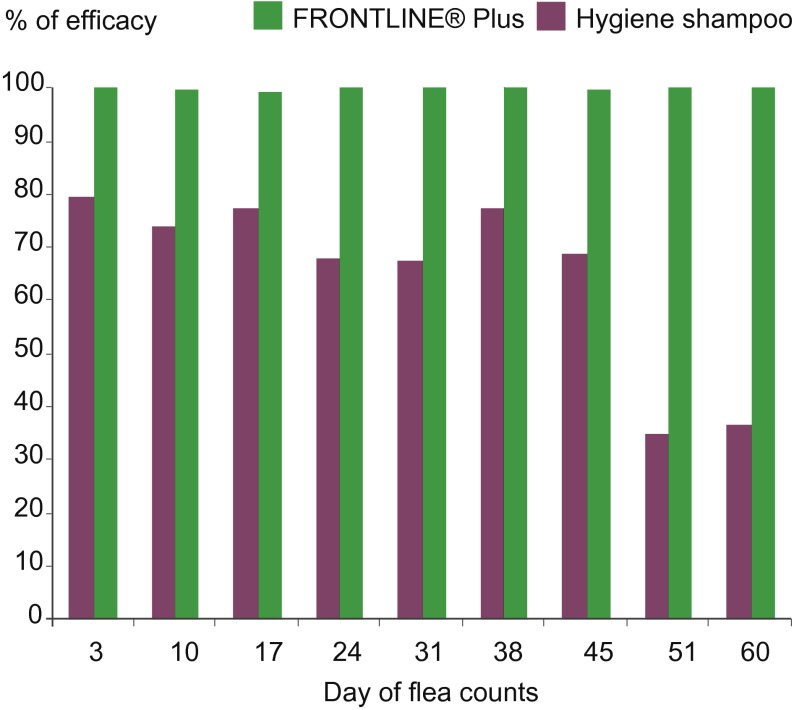
Adult flea efficacy of the two treatment protocols: eight weekly shampoos compared to two spot-on applications one month apart.

The average number of fleas counted on dogs was significantly different (p < 0.05) between the untreated and the two treatment groups and between the two treatment groups at all counts throughout the twomonth study (Days 3, 10, 17, 24, 31, 38, 45, 51 and 60) (Table 2, Fig. 4). The efficacy was above 99.1% in the fipronil/(S)-methoprene group, and efficacy in the shampoo group was never above 79.2%.

Weekly shampooing in treatment group 3 was intentionally delayed after Day 42 to demonstrate whether missing a weekly shampooing would affect the flea population. The Day 48 data indicated that forgetting or delaying a single weekly shampooing resulted in a clear increase in flea numbers and a dramatic decrease in the efficacy of regular shampooing from 68.2% to 34.8% (significant at p < 0.05 using a parametric paired-t-test).

**Fig. 5.**
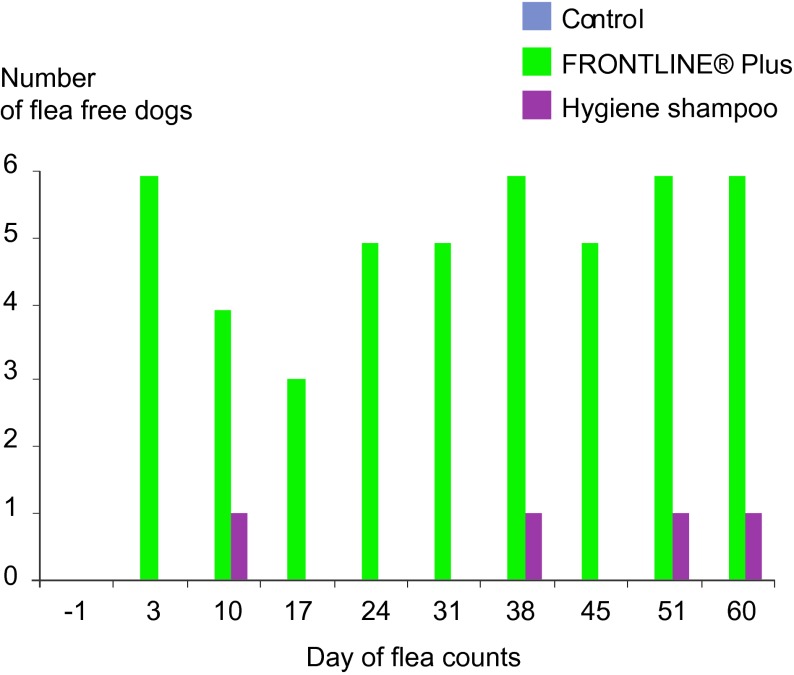
Number of flea free dogs (n = 6) during the 60 days of the study.

**Fig. 6.**
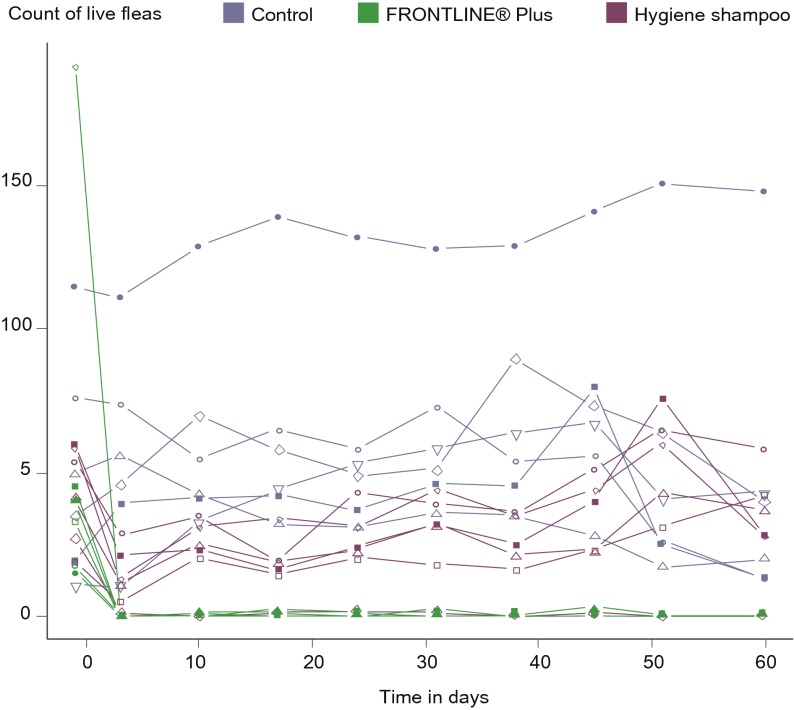
Plot of the number of live fleas per dog. Lines represent data observed in the same dog.

The fipronil/(S)-methoprene treatment allowed better continuous control as shown by the number of fleafree dogs, which was significantly different (p < 0.05, Fisher test) at all assessed time points, except Days 10 and 17 (Figs 5, 6). Despite the continuous heavy flea challenge in the dog’s environment, the number of flea-free dogs ranged from 3 to 6 (50 to 100%) of the six dogs in the fipronil/(S)-methoprene group, whereas there was never more than one flea-free dog (16.67%) in the shampoo group at any time point.

## Discussion

This study was designed to simulate the level of challenge presented in a natural, home flea infestation. In addition to the pre-infestations of adult fleas on Days -21 and -28, by the weekly placement of flea pupae in the specially-designed plastic container built into the roof of each sleeping crates, investigators were able to maintain infestations on the study dogs. Other authors have attempted to simulate the contamination seen in home infestations with the most successful animal models being developed using cats (Franc *et al.*, 2003; Jacobs *et al.*, 2001; Bradbury & Lappin, 2010). Considering the average flea infestation observed in the control group throughout the 60 days study, we consider the current design to be successful.

Regular weekly shampooing of dogs did reduce the number of fleas compared to the controls, but it was not sufficient to eliminate flea burdens as shown by the fact that only one dog in the shampoo group was free of fleas at only four of the nine assessments during the two month study. All other dogs from this group had from 5 to 60 fleas on every count, and each maintained consistent exposure rates throughout the study. The maximum overall reduction in flea number was never above 79.2% in the shampoo group, which does not meet from the efficacy guidelines required by many Regulatory agencies like EMA and WAAVP to get a flea claim (> 95%). This moderate reduction seen following regular weekly shampooing in this controlled study resulted from properly bathing the entire dog, using the appropriate quantity of shampoo and water, using experienced and trained laboratory personnel. Such a level of consistency and performance would not be replicated typically by the dog owners. The impact of inadequate shampooing was demonstrated clearly when weekly baths were delayed, simulating ”missing” a week, and the resultant flea counts subsequently increased. Control of continuous flea infestations provided by the fipronil/(S)-methoprene treatment was seen within the first week, and control was sustained throughout the study (Figs 4, 5, 6). Efficacy stayed above the threshold required by WAAVP guidelines, and the highest levels of 99.1% to 100% efficacy were achieved in four of the nine counts. Based on these results, weekly bathing using hygienic shampoos to eliminate fleas would not be expected to be effective under the continuous challenge seen in an infected home environment. The control of flea populations under natural challenge conditions, such as those seen in a pet owner’s home, are best achieved by using effective, long acting ectoparasiticides, such as the combination of fipronil/(S)-methoprene (Beugnet & Franc, 2009; Young *et al.*, 2004). This combination previously was shown to be able to provide good control of fleas even under high challenge conditions, such as those found in Tampa Florida, USA (Dryden, 2000; Dryden, 2011), and other similar warm, humid environments. It is the opinion of the authors that hygienic shampoos are best used for their intended objective, which is to clean the animal.

FRONTLINE^®^ is a registered trademark of Merial. All other marks are the property of their respective owners.
